# A looming epidemic: combating the recurrent outbreaks of diphtheria in Nigeria

**DOI:** 10.11604/pamj.2023.45.186.41328

**Published:** 2023-08-28

**Authors:** Olufemi Nicholas Olulaja, Emmanuel Temitope Anjorin, Olabode Ekerin, Oluwatoyosi Tolulope Afolabi, Jemimah Mayowa Inuojo

**Affiliations:** 1 Department of Public Health, School of Health Studies, College of Health and Human Sciences, Northern Illinois University, DeKalb, Illinois, United States,; 2 Department of Medicine and Surgery, Lagos State University Teaching Hospital (LASUTH), Ikeja, Lagos, Nigeria,; 3 Department of Population and Reproductive Health, School of Public Health, University of Port Harcourt, Rivers State, Nigeria,; 4 Xcene Research (Contract Research Organization (CRO)), Ikeja, Lagos, Nigeria

**Keywords:** Diphtheria, prevention, control, Nigeria

## Abstract

Nigeria has endured several diphtheria outbreaks over the last few decades, mirroring a suboptimal population immunity across several demographics within the country. The country's northern region has been affected mainly by this infectious disease; it directly depicts the effect of poor DPT vaccine uptake amongst children in this region compared to other geopolitical zones in Nigeria. Whilst pharmaceutical intervention and surveillance activities have commenced as directed by the NCDC, to combat this public health menace, top leaders of the Nigerian healthcare system - public and private sectors, must understudy the predisposing factors gearing the recurrence of diphtheria in Nigeria and provide robust, research-based and scientific mechanisms to arrest the root causes of the incessant outbreaks. This article discusses the factors promoting the recurrent diphtheria outbreaks in Nigeria, the preexisting interventions with their existential deterrents, and new strategies recommended to curb the further resurgence of the disease.

## Essay

**Figure 1:**
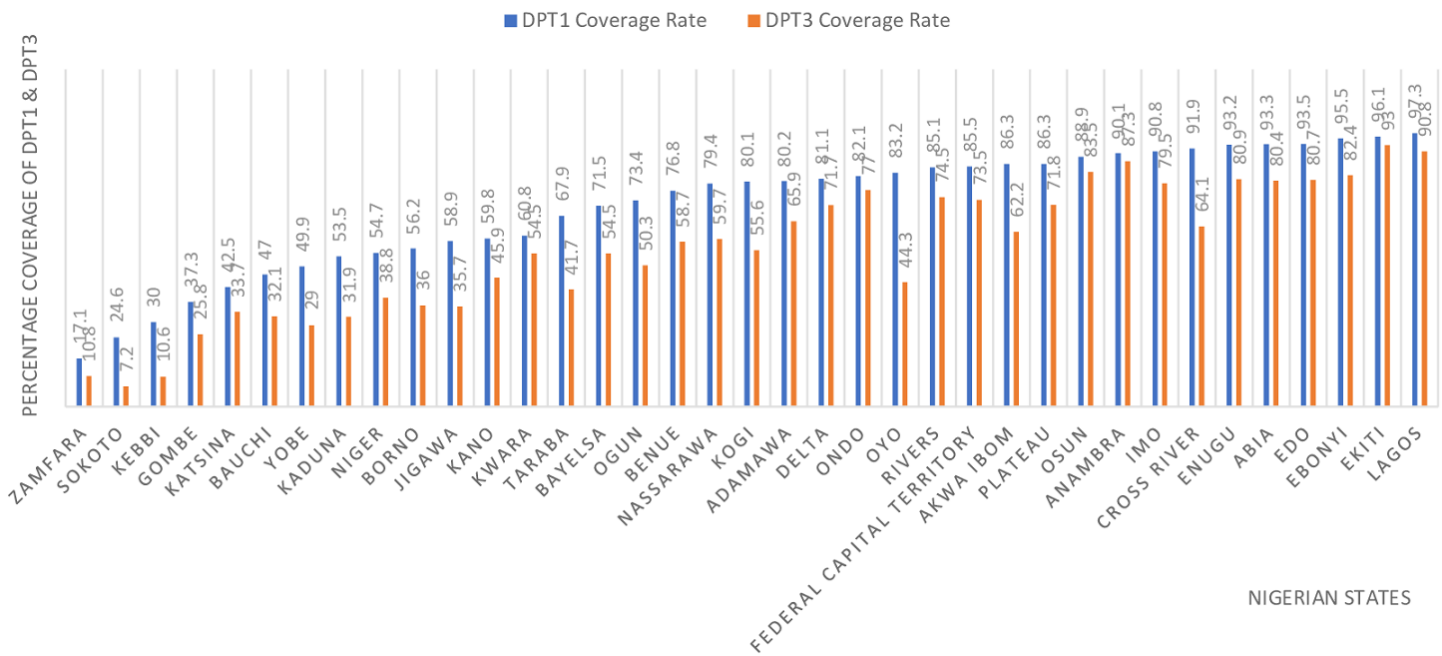
DTP (diphtheria, tetanus, pertussis) vaccination coverage in Nigeria

As of June 30, 2023, the Nigerian Center for Disease Control and Prevention (NCDC) had confirmed 798 cases of diphtheria across 33 Local Government Areas (LGAs) in eight States within Nigeria [1]. Of these cases, 782, representing approximately 98%, were from Kano State alone. Lagos, Yobe, Katsina, Cross River, Kaduna, and Osun contributed to the remaining figure [1]. Approximately 72% of cases were among children aged two to fourteen years, with 80% of the total cases resulting in mortality and a fatality rate of 10% [1]. Diphtheria is an acute infection caused by the rod-like, exotoxin-producing, gram-negative bacteria *Corynebacterium diphtheriae* [2]. It is highly contagious and spreads through respiratory droplets or direct contact [3]. Some symptoms include sore throat, fever, barking cough, enlarged lymph nodes, difficulty swallowing, and airway blockage [3]. Myocarditis can occur in 25% of cases, including affectation of the peripheral nervous system and temporary paralysis [4]. The treatment plan for diphtheria usually involves antibiotics and diphtheria antitoxins [3].

Despite diphtheria being a toxic infection, it is vaccine-preventable. The Diphtheria vaccine is in the National Programme on Immunization (NPI) schedule and available in the DTP (diphtheria, tetanus, pertussis) vaccine administered to neonates at 6, 10, and 14 weeks of life. The three doses of the diphtheria toxoid vaccine provide 87% protection against symptomatic disease and reduce transmission by 60% [5]. The Nigeria immunization schedule program has faced challenges of moderate to low coverage since its existence over 30 years ago [6]. The Nigeria Multiple Indicator Cluster Survey (MICS) and National Immunization Coverage Survey (NICS) showed a third dose of pentavalent coverage of 57% in 2021 [7]. Despite this being an improvement from the 33.3% coverage recorded in the 2016-2017 report [8], the increasing number of cases indicates the need to improve vaccine coverage. The 2021 MICS/NICS report revealed that low figures for third-dose pentavalent coverage are worse in the northern part of the country. The insecurity in the northeastern part of the country has contributed significantly to the low coverage in the region [3]. The World Health Organization (WHO) recommends prioritizing high-risk populations, such as under-five children, people in close contact with diphtheria cases, school-aged children, and healthcare personnel, for diphtheria vaccination [3].

In light of the recent outbreak, the NCDC has taken measures to halt the spread of the infection. The efforts started with the creation of a multi-sectoral National Diphtheria Technical Working Group to organize nationwide surveillance and response activities [1]. Some of these activities include the creation of Rapid Response Teams (RRT), the provision of diphtheria antitoxins, the development of Standard Operating Procedures (SOPs), sensitization and clinical training of surveillance officers, capacity building for public health laboratories, etc. [1]. While these measures are a step in the right direction, it is crucial to study and examine the underlying systemic factors responsible for the increasing resurgence of diphtheria within the country and recommend appropriate strategies to prevent future outbreaks by implementing continuous and coordinated epidemiological surveillance and response systems.

### Drivers of diphtheria outbreaks

Diphtheria, once a global public health threat, has experienced a decline in numerous countries due to the implementation of immunization initiatives and improved medical interventions. Despite being a vaccine-preventable disease, Nigeria continues to grapple with the re-occurrence of outbreaks. The persistence of diphtheria epidemics in Nigeria necessitates a more comprehensive examination of the underlying factors contributing to its resurgence.

***Vaccine coverage and immunization:*** the administration of diphtheria vaccination is an integral component of routine immunization protocols in Nigeria; nevertheless, specific regions exhibit suboptimal coverage rates. States in northern Nigeria consistently have low vaccine coverage rates for DPT1 and DPT3 [9] (Figure 1). The presence of disparities in vaccination coverage across different regions and demographic groups within Nigeria serves as a contributing factor to the occurrence of diphtheria outbreaks [10]. Inadequate vaccine coverage can result in diminished population immunity and consequent susceptibility of such regions to disease outbreaks. Even individuals who received childhood vaccinations may experience a waning of their immune protection over time, rendering them susceptible to illness [10]. Sub-optimal vaccine coverage contributes to gaps in population immunity, thereby facilitating disease transmission to more susceptible individuals [11].

***Vaccine hesitancy:*** driven by misinformation and mistrust in vaccination [12], is a crucial concern worldwide. It poses a formidable barrier to achieving herd immunity and curtailing disease transmission. A study in Northern Nigeria revealed that vaccine hesitancy is influenced by knowledge, behavior, and perception of vaccination [12]. Also, the literacy rate may be a significant contributor to vaccine hesitancy. Figure 1 shows the relationship between literacy rate and vaccine coverage. It reveals that regions in northern Nigeria with the lowest literacy have low vaccine coverage for DPT1 and DPT3. Insufficient public awareness about diphtheria and its prevention hampers early recognition of symptoms and delays seeking medical care. This may affect DPT uptake and exacerbate the diphtheria epidemic, hindering progress in achieving herd immunity.

***Conflicts and insurgencies:*** regions affected by conflict and insurgencies exhibit disparate levels of access to immunization programs [13], resulting in the emergence of vulnerable populations that facilitate disease transmission. The northern part of Nigeria is greatly affected by different forms of unrest in the form of terrorism, banditry, and religious crisis. These parts of the country are also the areas where we have low vaccine coverage. Conflicts and insurgencies influence migration patterns, thereby disrupting established health infrastructures.

***Health system challenges:*** Nigeria's healthcare system faces the challenges of inadequate resources, facilities, and personnel [14]. The occurrence of diphtheria outbreaks is contingent upon the healthcare system's capacity to provide effective treatment to affected individuals. A weak health system may result in uncontrolled transmission of diphtheria [11]. The potential outcomes of delayed treatment can be severe and increase mortality rates. The limited availability of accessible healthcare facilities can exacerbate disease transmission, severity of illness, and development of complications.

**Figure 2:**
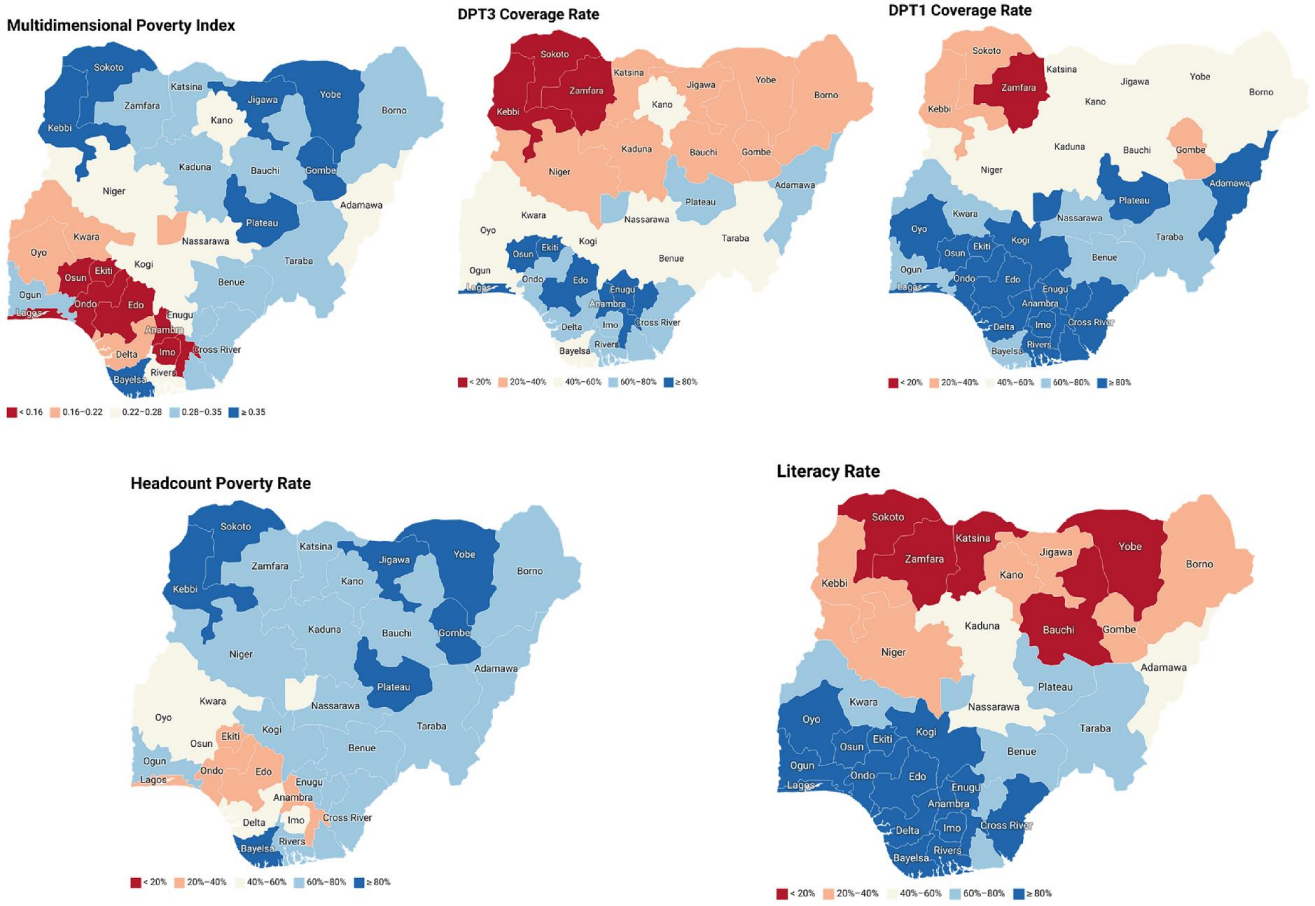
overview of multidimensional poverty index (MPI), headcount poverty rate, and literacy rates in Nigeria

***Socioeconomic and cultural factors:*** the dynamics of infectious diseases are significantly influenced by socioeconomic determinants [15]. Socioeconomic disparities among individuals residing in various states within Nigeria may contribute to the difficulties encountered in disease prevention and control, including diphtheria. States in northern Nigeria with high multidimensional poverty index (MPI) and high headcount poverty rates have low vaccine coverage (Figure 2) [16,17]. The MPI and headcount poverty rate may impact individuals' healthcare-seeking behavior and adherence to vaccination schedules. The transmission of infectious diseases is intensified by factors such as poverty, malnutrition, restricted healthcare accessibility, inadequate sanitation, and densely populated living environments [18]. Insufficient vaccination rates, particularly within socioeconomically disadvantaged populations, give rise to localized clusters of vulnerable individuals, thereby facilitating disease transmission.

***Surveillance and reporting:*** accurate and timely surveillance is essential for disease monitoring and response. Delays in reporting cases, as well as underreporting, can impede effective outbreak containment [19]. Effective disease surveillance and reporting systems are crucial for early detection and containment of diphtheria outbreaks. However, Nigeria's healthcare system may face shortcomings in promptly identifying and reporting cases, which can lead to delays in implementing control measures. Challenges in promptly diagnosing diphtheria cases due to limited laboratory facilities, inadequate sample transportation, and lack of diagnostic expertise can lead to delayed response measures, allowing the disease to spread further.

***Pathogen dynamics and antimicrobial resistance:*** the emergence of antimicrobial-resistant strains of *Corynebacterium diphtheriae* presents a grave challenge to outbreak control efforts [20]. Also, a deep understanding of the genetic diversity and evolution of *C. diphtheria* strains circulating in Nigeria is vital for comprehending outbreak patterns. Pathogen genetic factors influence strain virulence, antimicrobial resistance, and transmission dynamics. The misuse and overuse of antibiotics in Nigeria contribute to the development and dissemination of resistant strains. Limited access to appropriate antibiotics and indiscriminate use of antimicrobials all contribute to the challenge of managing diphtheria cases.

### Strategies to combat recurrent diphtheria outbreaks

Given the recent recurrent outbreaks of diphtheria in Nigeria, it is imperative to develop evidence-based strategies to reduce transmission and deaths in the short term while also defeating the infection in the medium to long term (Table 1). Nigeria must stop the outbreak and prevent morbidities and deaths due to diphtheria infection in the short term. In the long term, there is a need to develop a framework capable of preventing future outbreaks.

**Table 1:** short and long-term strategies to combat diphtheria outbreaks

Short term	Long term
Public awareness and education	Improved funding for immunization programs
Improved clinical suspicion and case management	Improved disease surveillance infrastructure
Improved medical supply chain	Improved access to care
Improved laboratory capacity	Government and organizational policies should promote vaccine uptake
Increased diphtheria vaccine coverage	

### Short-term strategies

***Public awareness and education:*** the current public awareness of diphtheria is suboptimal as it still needs to be discovered by many Nigerians, especially rural dwellers who are even at greater risk. Nigeria's robust public awareness of COVID-19 was central to the containment of the SARS-CoV-2 virus, with social media being the most effective [21]. The level of public awareness of diphtheria should be at par with that of COVID-19 for there to be a significant impact. Public awareness should be culturally responsive and available in different Nigerian languages across the most widely used media platforms (radio, newspapers, awareness walks, campaigns, and social media). The modality may include house-to-house campaigns, rallies, posters, TV shows, radio programs, daily/weekly incidence reports, social media engagements, etc. The goal here is to sensitize the public about the current outbreak, discuss early symptoms, increase the suspicion level of community residents, and publicize the need for early presentation at the nearest health facility.

***Improved clinical suspicion and case management:*** according to the World Health Organization (WHO), clinical diagnosis alone is sufficient to commence diphtheria management [22]. Treatment should be instituted while awaiting laboratory diagnosis. High-level clinical suspicion of diphtheria based on presenting signs and symptoms is essential to reduce poor outcomes and prevent deaths among people infected with diphtheria. Once there is a suspicion of diphtheria infection, initial clinical management includes isolation of the suspected case, prompt administration of diphtheria antitoxin (DAT) and antibiotics, close monitoring, and supportive therapy (airway management, neurologic, cardiac, and renal support), and age-appropriate diphtheria toxoid vaccination. Appropriate guidelines, management protocols, and standard operating procedures will help provide quality care. Furthermore, in rural settings where Community Health Officers (CHOs) and Community Health Extension Workers (CHEWs) provide frontline care [23], the NCDC should conduct adequate training to improve capacity for clinical suspicion and prompt referral to bigger facilities.

***Improved medical supply chain:*** diphtheria antitoxin (DAT), the mainstay of short-term disease management, is not readily available in all parts of Nigeria. If administered within the first hour of clinical diagnosis, DAT may reduce mortality to 1% compared to 20% on the fourth day [24]. Among the ninety-eight (98) cases that met the case definition for diphtheria during the 2011 outbreak in Borno State, none received diphtheria antitoxin because it was unavailable [25]. Very recently, the Nigerian Center for Disease Control and Prevention (NCDC), with the support of the WHO, procured DAT to distribute among affected states. Adequate stocking of DAT should be prioritized at this time with unhindered availability in all parts of the country, not only the currently affected states. Consumables such as reagents, sample collection kits, etc. should be in sufficient quantity.

***Improved laboratory capacity:*** laboratory capacity for diagnosing diphtheria is low in Nigeria; hence most cases are treated based on clinical suspicion. Definitive diagnosis of diphtheria is performed by laboratory isolation of etiologic pathogen (speciation and antibiotic susceptibility) and differentiation of toxigenic from non-toxigenic strains. It is imperative to improve the capacity of States and other reference laboratories to provide a definitive diagnosis of diphtheria. Before the COVID-19 pandemic, only a few Nigerian laboratories could perform polymerase chain reaction (PCR), but this capacity improved significantly in response to the pandemic. A similar approach should be deployed to defeat diphtheria, with the Nigerian Center for Diseases Control and Prevention (NCDC) providing the technical expertise required to improve nationwide laboratory testing of diphtheria. Training physicians, laboratory scientists, and technicians become essential in this context. Relevant protocols and standard operating procedures will reinforce standardization and quality.

***Increased diphtheria vaccine coverage:*** in the ongoing outbreak, as of July 6, 2023, 82% of the confirmed cases were unvaccinated individuals [1]. Nigeria's diphtheria vaccine coverage is suboptimal, with overall coverage at 57% [26]. This low coverage undermines efforts to control the infection of a vaccine-preventable disease effectively. Vaccination should be improved across all states, focusing on rural communities and northern parts of the country where cases are predominant. Booster doses should also be encouraged. To improve vaccination coverage, create public awareness of the vaccine's importance while addressing misinformation about the DTP vaccine.

### Long-term strategies

***Improved funding for immunization programs:*** Nigeria needs to increase its funding for immunization programs. The budget should be directed toward raising public awareness of vaccine availability, distributing vaccines to hard-to-reach communities, improving the vaccine supply chain, etc.

**Figure 3:**
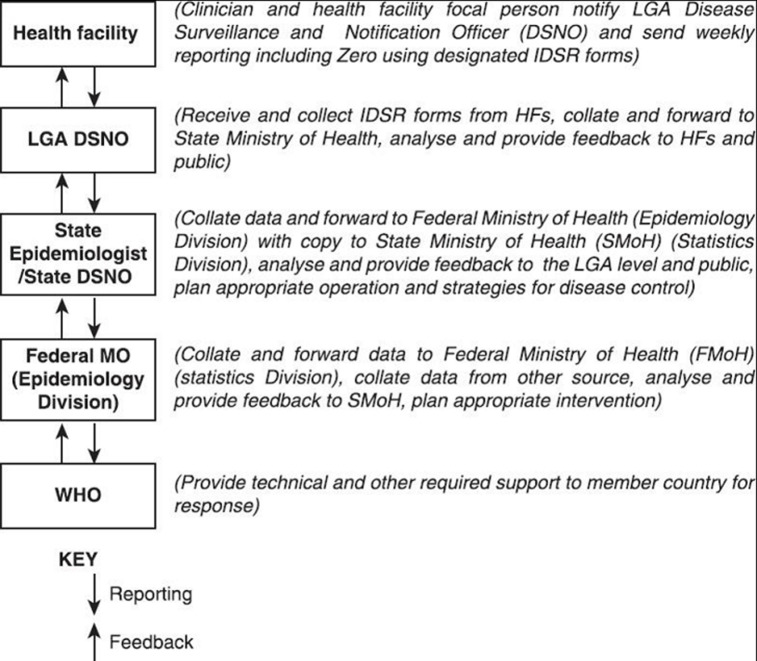
flow of Integrated Disease Surveillance and Response (IDSR) in Nigeria

***Improved disease surveillance infrastructure:*** although there is a clearly described disease notification process for diphtheria in Nigeria, all cases must be reported promptly. According to the World Health Organization (WHO), the reporting of diphtheria cases in Nigeria has witnessed constant dwindling from 5039 (in 1989) to 2468 (in 2001) and further decline to 312 (2006), with no data available for some years after 2006 [6]. This may be due to inefficient surveillance infrastructure and poor reporting [6]. The disease notification framework (Figure 3) [27] developed by the Federal Ministry of Health (FMoH) should be strictly adhered to improve the surveillance system. The FMoH should democratize data by making it accessible to all. This will encourage research and improve the quality of scientific evidence on disease prevalence, vaccination coverage, etc.

***Improved access to care:*** overall, improved access to care will be central to combating diphtheria outbreaks. Most of the recent cases are among unvaccinated individuals, with Northern Nigeria recording most of the cases. Coincidentally, the northern parts of Nigeria experience reduced access to care compared to the south. As such, the high incidence of diphtheria in the north may reflect its reduced healthcare access.

***Government and organizational policies should promote vaccine uptake:*** this was effective during the COVID-19 pandemic when governments and organizations formulated policies encouraging vaccine uptake. For example, the government may require parents to present vaccination updates of their children as a requirement during school enrolment. Parents whose children have incomplete vaccine records should be counseled on the need for vaccines and directed to appropriate centers for vaccination. Creche owners can promote a similar policy during enrolment in a creche. This may encourage vaccine uptake and consequently improve vaccine coverage.

## Conclusion

Curbing recurrent diphtheria outbreaks is possible through carefully implemented evidence-based public health interventions channeled towards addressing the primary factors influencing these outbreaks. A painstaking effort to implement the practical approach contained in this paper would dwindle the incidence rate over the years, and then put an absolute end to diphtheria outbreaks in Nigeria.
